# Pathogenicity potential of enterococci isolated from a Veterinary Biological Isolation and Containment Unit

**DOI:** 10.3389/fvets.2024.1458069

**Published:** 2024-10-21

**Authors:** Catarina Geraldes, Catarina Araújo, Ana Catarina Pinheiro, Mónica Afonso, Sandra Carapeto, Cláudia Verdial, Eva Cunha, Raquel Abreu, Luís Tavares, Lélia Chambel, Solange Gil, Manuela Oliveira

**Affiliations:** ^1^CIISA - Centre for Interdisciplinary Research in Animal Health, Faculty of Veterinary Medicine, University of Lisbon, Lisbon, Portugal; ^2^AL4AnimalS - Associate Laboratory for Animal and Veterinary Sciences, Faculty of Veterinary Medicine, University of Lisbon, Lisbon, Portugal; ^3^Faculty of Veterinary Medicine, University of Lisbon, Lisbon, Portugal; ^4^Department of Veterinary and Animal Sciences, University of Copenhagen, Copenhagen, Denmark; ^5^BioISI - Biosystems and Integrative Sciences Institute, Faculty of Sciences, University of Lisbon, Lisbon, Portugal; ^6^BICU - Biological Isolation and Containment Unit, Veterinary Teaching Hospital, Faculty of Veterinary Medicine, University of Lisbon, Lisbon, Portugal; ^7^cE3c – Centre for Ecology, Evolution and Environmental Changes and CHANGE—Global Change and Sustainability Institute, Faculty of Sciences, University of Lisbon, Lisbon, Portugal

**Keywords:** *Enterococcus*, hospital-acquired infections, antibiotic resistance, virulence, veterinary, Biological Isolation and Containment Unit

## Abstract

**Introduction:**

*Enterococcus* are considered an important genus in terms of Hospital-Acquired Infections (HAIs), which means that their characterization regarding resistance and virulence profiles in the hospital environment is of extreme importance. This article addresses this issue through the characterization of enterococci collected from a Veterinary Biological Isolation and Containment Unit (BICU).

**Methods:**

A total of 73 isolates, collected from different surfaces of a Veterinary BICU, were identified as *Enterococcus* through PCR at species level, after which 34 isolates were selected as representatives using (GTG)_5_ fingerprinting. These isolates were further characterized phenotypically in terms of antimicrobial resistance through disk diffusion and of virulence factors’ expression.

**Results:**

The majority of the enterococci isolated presented resistance to erythromycin (79.4%), ampicillin (73.5%), amoxicillin-clavulanic acid (70.6%), tetracycline (67.6%), ciprofloxacin (58.8%) and levofloxacin (50.0%), and were able to produce hemolysin (88.2%) and biofilm (82.3%). Furthermore, in terms of pathogenicity, three isolates (8.8%) were classified as high threats and two (5.9%) as moderate threats.

**Discussion:**

The degree of resistance, production of virulence factors and the percentage of isolates classified as moderate or high threat means that a constant vigilance of such strains in veterinary units, but also in clinics and hospitals in general, is an important tool in terms of infection prevention and consequent reduction of HAIs.

## 1 Introduction

Enterococci, particularly *Enterococcus faecalis* and *E. faecium*, have gained significant attention due to their emerging role in Hospital-Acquired Infections (HAIs) within human and veterinary settings. According to the last European Centre for Disease Prevention and Control (ECDC) report, enterococci accounted for 18.9% of intensive care unit (ICU)-acquired bloodstream infections and 23.0% of ICU-acquired urinary tract infections in patients admitted to ICUs in 2020, being the second most prevalent bacteria in both cases (1). Additionally, another report by the ECDC referred that, between 2018 and 2020, 17.6% of surgical site infections were cause by *Enterococcus* spp., only surpassed by *Staphylococcus* spp. (29.2%) (2).

Not being highly virulent organisms, their capacity to cause infection mainly derives from: (i) their ability to linger in the environment for long periods of time due to their biofilm-forming capacity (3); (ii) their reduced susceptibility to many antimicrobial agents such as *β*-lactams, aminoglycosides, and glycopeptides (3–6); (iii) and the plasticity of their genome, which allows them to easily acquire, conserve and disseminate genetic traits, not only among enterococci but also to other Gram-positive bacteria (3, 7–10). Therefore, preventing the existence and permanence of these microorganisms in the hospital’s and ICU’s environment is an essential practice to reduce the number of HAIs (11).

In veterinary practice, Biological Isolation and Containment Units (BICUs) correspond to the hospital areas where animals are placed to mitigate the spread of infections to other animals or humans (zoonosis). These units are preferably located in a distinct area of the hospital, usually with a separate entrance, properly identified and with restricted access only to staff essential for the treatment of the hospitalized animals. These areas should always have their own standard operation procedures (SOPs), available to all personnel (12). If possible, they should also be under negative pressure (<2.5 Pa), with 6 to 12 air changes per hour, to contain all pathogenic organisms present in the air, exhausting them directly to the exterior; if not, a high-efficiency particulate air (HEPA) filter should be installed (13, 14). Other procedures, such as limitation of owner visits, adequate use of personal protection equipment (PPEs), and rigorous cleaning and disinfection protocols should also be applied in those settings (12, 15).

In this study, *Enterococcus* isolates were collected from the surfaces of a BICU belonging to a Veterinary Teaching Hospital in two distinct periods of time, after which they were identified and their antimicrobial susceptibility and virulence profiles determined, aiming to evaluate a high-risk transmission scenario. Taking into account enterococci’s environmental permanence and their importance in terms of HAIs, combined with their intrinsic resistance to many antimicrobials, these bacteria could become an important source of infection to animals in a BICU, and their presence should consequently be assessed, not only in these units but also in veterinary hospitals and clinics.

## 2 Materials and methods

### 2.1 Description of the Biological Isolation and Containment Unit of the Veterinary Teaching Hospital

The BICU of the Veterinary Teaching Hospital (Faculty of Veterinary Medicine, University of Lisbon) is located in a building separate from the hospital, with its own entrance and restricted access. It receives companion animals suspected or confirmed of having an infectious disease, most commonly upper respiratory infections (URIs), FeLV, FIV, and panleukopenia in the case of cats (16), and parvovirus, leptospirosis, multi-drug resistant (MDR) infections, and distemper in the case of dogs (17).

The unit is divided into a total of four hospitalization rooms: one for cats, two for dogs, and one for intermedium cat patients, i.e., for those suspected but not confirmed of having an infectious disease due to the lack of vaccination or testing, or for cats infected with retrovirus but with no other concomitant diseases. There is also a room that works as a reception and consulting room for the animals that are admitted to the BICU, a work room for medical personnel and students, a preparatory room, and a storage room.

This unit has implemented several infection control measures. These include the presence of a ventilation system equipped with a HEPA filter that maintains a stable temperature and negative pressure, the availability of several SOPs that, amongst others, give indications on how to manipulate patients (including utilization of PPEs), on hand hygiene and also on cleaning and disinfecting protocols for different BICU areas, including daily cleaning and disinfection of the floors and surfaces, weekly cleaning and disinfection of each room and a yearly fumigation of the entire unit, independently of the number of animals housed in the area.

All hospitalization rooms are equipped with cages and examination tables made of stainless steel, a cabinet for material storage needed for patient examination (each room has its own materials), and a sink.

### 2.2 Sampling procedures

**Figure 1:**
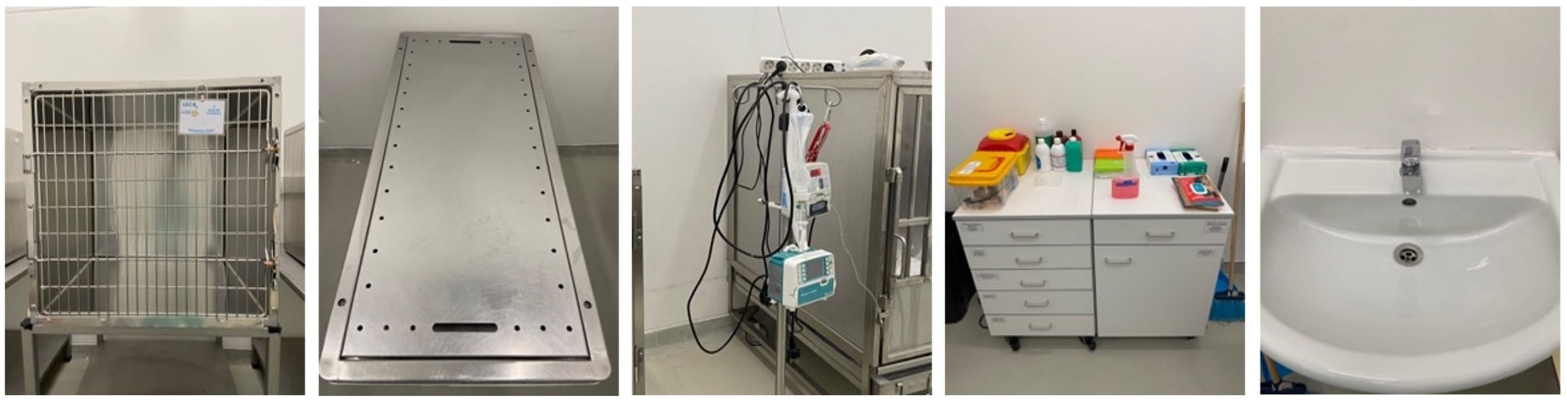
Examples of different surfaces sampled in this study.

A total of 200 samples were obtained between February and July of 2022 from different high-critical surfaces of the BICU: cages (*n* = 80), cabinets (*n* = 41), examination tables (*n* = 39), effusion pumps (*n* = 18), door handles (*n* = 10), sinks (*n* = 8), incubator (*n* = 2), and of the plastic box used to storage examination material (stethoscope, thermometer, among others) (*n* = 2) (Figure 1).

Samples were collected using a swab embedded in a saline solution. Cages were sampled when fully disinfected and ready to receive new patients, but also when the disinfection process was still underway, since these surfaces could serve as a source of cross-contamination. A total of 52 samples were collected from fully disinfected cages, while 9 samples were taken on the first 24 h (first day), 12 on between 24 and 48 h (second day) and 7 between 48 and 72 h (third day) after disinfection.

Collected swabs were immediately transported to the Laboratory of Bacteriology of the same Faculty. After vortex homogenization, 100 μL of each sample were inoculated in Slanetz Bartley agar (AppliChem, Darmstadt, Germany) with the aid of sterile glass beads, and incubated at 36°C (±1°C) for 48 h. Subsequently, four colonies with macroscopic characteristics compatible with *Enterococcus* spp., i.e., red, brown or pink colonies, were collected and further characterized through Gram staining, catalase reaction, and the bile esculin test (18). Finally, the presumptive identification of all enterococci was confirmed through PCR.

An additional 23 presumptive *Enterococcus* isolates collected from surfaces of the BICU in 2019 by Verdial et al. (19), and previously reported in another study (20), were also added to this collection and also identified by PCR.

### 2.3 PCR identification

#### 2.3.1 Genus identification

Genus identification was made using primers and adapted conditions as described by Ke et al. (21), in mixtures containing a total of 25 μL composed of: 12.5 μL of Supreme NZYTaq II 2x Green Master Mix (NZYTech, Lisbon, Portugal), 1 μL of DNA and 1 μM of each Ent1 and Ent2 primer (Table 1). Positive (*E. faecalis* ATCC® 29212) and negative (sterile PCR water) controls were included in all reactions. Thermocycler conditions were as follows: an initial step of 94°C for 3 min, 35 cycles with 1 min at 94°C, 1 min at 48°C, and 1 min at 72°C, followed by a final step at 72°C for 5 min. PCR products were evaluated by agarose gel electrophoresis (1.3%, w/v) in 1x TBE buffer (NZYTech, Lisbon, Portugal) supplemented with GreenSafe Premium (NZYTech, Lisbon, Portugal) at 90 V for 1 h, and photographed with the ChemiDoc™ Gel Imaging System (Bio-Rad, California, United States).

**Table 1:** Primers used for genus and species identification.

Identification	Primer		Product length	Reference
*Enterococcus* spp.	Ent1	5’ TAC TGA CAA ACC ATT CAT GAT G 3′	112 bp	Ke et al. (21)
	Ent2	5’ AAC TTC GTC ACC AAC GCG AAC 3′		
*Enterococcus faecium*	FM1	5′ GAA AAA ACA ATA GAA GAA TTA T 3′	215 bp	Jackson et al. (44)
	FM2	5’ TGC TTT TTT GAA TTC TTC TTT A 3’		
*Enterococcus faecalis*	FL1	5’ ACT TAT GTG ACT AAC TTA ACC 3’	360 bp	Jackson et al. (44)
	FL2	5′ TAA TGG TGA ATC TTG GTT TGG 3’		
*Enterococcus hirae*	HI1	5’ CTT TCT GAT ATG GAT GCT GTC 3’	187 bp	Jackson et al. (44)
	HI2	5′ TAA ATT CTT CCT TAA ATG TTG 3’		
*Enterococcus durans*	DU1	5’ CCT ACT GAT ATT AAG ACA GCG 3’	295 bp	Jackson et al. (44)
	DU2	5′ TAA TCC TAA GAT AGG TGT TTG 3’		
Fingerprinting	(GTG)_5_	5′ GTG GTG GTG GTG GTG 3’	200–3,000 bp	Semedo-Lemsaddek et al. (23)

#### 2.3.2 Species identification

Species identification was made through a multiplex PCR for *E. faecium*, using primeirs FM1 and FM2, and *E. faecalis*, using primers FL1 and FL2, and for *E. hirae*, using primers HI1 and HI2, and *E. durans*, using primers DU1 and DU2, according to protocols adapted from Jackson et al. (22) (Table 1). Positive (*E. faecalis* ATCC® 29212, *E. faecium* CCUG® 36804, *E. hirae* ATCC® 10541, *E. durans* DSM® 20633) and negative controls (sterile PCR water) were included in all reactions, and independent replicas (10%) were also performed to guarantee the validity and reproducibility of the results.

For *E. faecium* and *E. faecalis* identification, PCR mixtures containing 12.5 μL of Supreme NZYTaq II 2x Green Master Mix (NZYTech, Lisbon, Portugal), 1 μL of DNA and 1.25 μM of each primer were prepared. Thermocycler conditions were as follows: an initial step of 95°C for 5 min, 35 cycles with 1 min at 95°C, 1 min at 54°C, and 1 min at 72°C, followed by a final step at 72°C for 10 min.

For *E. hirae* and *E. durans* identification, PCR mixtures containing 12.5 μL of Supreme NZYTaq II 2x Green Master Mix (NZYTech, Lisbon, Portugal), 1 μL of DNA and 0.75 μM of each primer were prepared. Thermocycler conditions were as follows: an initial step of 95°C for 5 min, 30 cycles with 30 s at 95°C, 1 min at 55°C and 1 min at 72°C, followed by a final step at 72°C for 7 min.

PCR products were evaluated as described before.

#### 2.3.3 Fingerprinting

The protocol used for genomic typing by fingerprinting was adapted from the one described by Semedo-Lemsaddek et al. (23), using primer (GTG)_5_ in mixtures containing: 1x reaction buffer, 3 μM MgCl_2_, 0.2 μM of each deoxynucleotide triphosphate (dNTP), 2 μM of Primer, 0.06 U of Taq (Invitrogen, Massachusetts, United States) and 100 ng of DNA. A negative control (sterile PCR water) was included in all reactions, and independent replicas (5%) were also performed to validate and evaluate the reproducibility of the results. Thermocycler conditions were as follows: an initial step of 94°C for 4 min, 40 cycles with 1 min at 94°C, 2 min at 40°C, and 2 min at 72°C, followed by a final step at 72°C for 10 min. PCR products were evaluated by agarose gel electrophoresis (1.5%, w/v) in 1x TBE buffer (NZYTech, Lisbon, Portugal) supplemented with GreenSafe Premium (NZYTech, Lisbon, Portugal) at 90 V for 70 min, and photographed with ChemiDoc™ Gel Imaging System (Bio-Rad Laboratories, California, United States).

The profiles were compared by BioNumerics® 6.6 (Applied Maths, Kortrijk, Belgium), with a hierarchical numerical process based on the Pearson correlation coefficient (optimization 0.5) and the unweighted pair group method with arithmetic average (UPGMA) as the agglomerative clustering. The reproducibility value was determined as the average value for duplicates, and a cut off of 70% was established.

Isolates that had a similarity above 70% but were from different years (2019 or 2022) were maintained for further testing.

### 2.4 Antimicrobial susceptibility testing

The characterization of the susceptibility profile of all isolates under study was performed through the disk diffusion method, made accordingly to the Clinical and Laboratory Standards Institute (CLSI) guidelines (24, 25). Control strains (*Staphylococcus aureus* ATCC® 25923 and *E. faecalis* ATCC® 29212) were also tested, as recommended by CLSI, and independent replicates (10%) were performed to assure the reproducibility of the results obtained.

The antibiotics to be tested were chosen according to their frequent use as a treatment option against enterococcal infections in both human and veterinary medicine: ampicillin (10 μg), amoxicillin and clavulanic acid (30 μg), ciprofloxacin (5 μg), levofloxacin (5 μg), tetracycline (30 μg), doxycycline (30 μg), erythromycin (15 μg), chloramphenicol (30 μg), linezolid (30 μg), vancomycin (30 μg), teicoplanin (30 μg), high-level gentamycin (120 μg) and high-level streptomycin (300 μg) (Oxoid Limited®, Hampshire, United Kingdom). High-level aminoglycoside testing in enterococci is performed to assess whether aminoglycosides can be used effectively in combination with penicillin or glycopeptides to treat infections caused by these bacteria. Additionally, if high-level resistance to both aminoglycosides is detected, it generally indicates that the bacteria are resistant to all aminoglycosides (24, 25).

Bacterial suspensions with turbidity equivalent to 0.5 in the McFarland scale (approximately 1.5 × 10^8^ CFU/mL) were prepared for all isolates using a Densimat® (bioMérieux, Lisbon, Portugal). These suspensions were then inoculated using the lawn technique on Mueller–Hinton agar (Oxoid Limited®, Hampshire, United Kingdom) plates, followed by placement of the antibiotic disc on the agar surface and incubation at 36°C (±1°C) for 18 h, or for 24 h in the specific case of vancomycin. The diameter of the zones of inhibition formed around the disc was measured and results were interpreted according to the CLSI guidelines M100 (24), VET09 (25), and M31-A3 (26).

The Multiple Antibiotic Resistance (MAR) index for each isolate was calculated according to Singh et al. (27): the number of antimicrobials to which isolates were resistant divided by the number of antimicrobials tested.

### 2.5 Virulence assays

Hemolysin, gelatinase, biofilm, DNAse, proteinase, and lecithinase production were evaluated phenotypically in different agar media, using the incubation conditions described by Fernandes et al. (28), using both positive and negative controls, as well as performing independent replicas (10%).

Hemolysin was evaluated on Columbia agar medium supplemented with 5% sheep blood (bioMérieux, Lisbon, Portugal). The formation of a transparent or greenish halo around the colonies was considered a positive reaction.

Gelatinase was evaluated in nutrient gelatin agar (Oxoid, Basingstoke, United Kingdom), using *Pseudomonas aeruginosa* ATCC® 27853 as a positive control and *Escherichia coli* ATCC® 25922 as a negative control. A liquefaction of the medium was classified as positive, after a 30-min refrigeration at approximately 4°C.

Biofilm was evaluated in Brain Heart Infusion agar (VWR, Leuven, Belgium) supplemented with Congo Red as an indicator (Sigma-Aldrich, St. Louis, United States) and sucrose (Merck KGaA, Darmstadt, Germany), using *E. faecium* ATCC® 29212 as a positive control and *Escherichia coli* ATCC® 25922 as a negative control. The formation of black colonies with a glossy surface was classified as positive.

DNAse was evaluated in DNAse agar (VWR, Leuven, Belgium) supplemented with toluidine blue (Merck KGaA, Darmstadt, Germany), using *S. aureus* ATCC® 25923 as a positive control and *Escherichia coli* ATCC® 25922 as a negative control. The formation of a pink halo around the colonies was considered a positive reaction.

Proteinase was evaluated in Skim Milk agar (VWR, Leuven, Belgium), using *P. aeruginosa* ATCC® 27853 as a positive control and *S. aureus* ATCC® 29213 as a negative control. The formation of a transparent halo around the colonies was considered a positive reaction.

Lecitinase was evaluated in Tryptic Soy Agar (VWR, Leuven, Belgium) supplemented with an egg yolk emulsion at 10% (VWR, Leuven, Belgium), using *P. aeruginosa* ATCC® 27853 as a positive control and *Escherichia coli* ATCC® 25922 as a negative control. The formation of a white precipitation halo around the colonies was considered a positive reaction.

#### 2.5.1 Biofilm quantification through crystal violet

Firstly, due to the lack of agreement regarding the ideal glucose supplementation value required to evaluate biofilm-forming ability by enterococci (29), a protocol was performed aiming to optimize glucose concentration, adapted from El-Zamkan and Mohamed (30) and Hashem et al. (29). Bacterial suspensions of approximately 1.5 × 10^8^ CFU/mL were prepared for three randomly chosen *Enterococcus* isolates, in 2 mL of Tryptic Soy Broth (TSB) (VWR, Leuven, Belgium) supplemented with various glucose concentrations (0.25, 0.5, 1.0 and 1.5%). This bacterial suspension was then further diluted 1:100 in equally supplemented TSB, after which 200 μL were inoculated onto 3 different wells of a 96-well plate. The same was performed for negative controls, which corresponded to solutions of the four mentioned different glucose concentrations without bacteria, and then the plate was incubated at 37°C (±1°C) during 48 h. After incubation, the contents of the wells were discarded, and the wells were washed three times with 150 μL of phosphate-buffered saline. The cells were then heat-fixed at 60°C for 1 h. After, 200 μL of crystal violet (Merck, Darmstadt, Germany) at 2% were added to each well, followed by a 15 min incubation at room temperature. Next, the non-adhered dye was removed by inverting the plate and then washing it with water. Finally, 200 μL of ethanol at 95% were added to each well, followed by a 30 min incubation at room temperature to resolubilize the dye. Optic density (OD) of each well was measured at 570 nm using the FLUOstar OPTIMA microtiter-plate reader (BMG Labtech, Ortenberg, Germany). This assay was repeated two more times in independent days.

Isolates were evaluated on their ability to form biofilm according to the OD value obtained as follows: strains with OD < ODc (average OD of negative controls) were considered as non-biofilm producers; those with ODc < OD < 2ODc were considered as weak biofilm producers; strains with 2ODc < OD < 4ODc were considered as moderate biofilm producers; and those with 4ODc < OD were considered as strong biofilm producers.

Biofilm production determination for all isolates was conducted using the same protocol, with a fixed glucose supplementation of 0.5% (final concentration of 0.75%).

The virulence index of each isolate was calculated as the sum of all positive virulence phenotypes exhibited by each isolate divided by the total number of virulence factors tested (27). Regarding biofilm formation capacity, only the results obtained by crystal violet staining were considered, since this is described as a more accurate method in comparison to Congo Red agar plate evaluation (29).

### 2.6 Statistical analysis

Fisher’s exact test and Pearson’s chi-squared were employed to investigate whether there were statistically significant differences in antibiotic resistance and the presence or absence of virulence factors between the isolates collected in the years 2019 and 2022. The *p*-values were calculated to assess the significance of the results, using a significance level of 0.05. RStudio (Integrated Development for R, Boston, United States of America), an integrated development environment for the R programming language, was utilized for performing the statistical analyses, using relevant packages (“dplyr,” “tidyr,” “reshape,” “tidyverse” and “ggplot2”) and functions as needed.

## 3 Results

### 3.1 Sample collection

**Figure 2:**
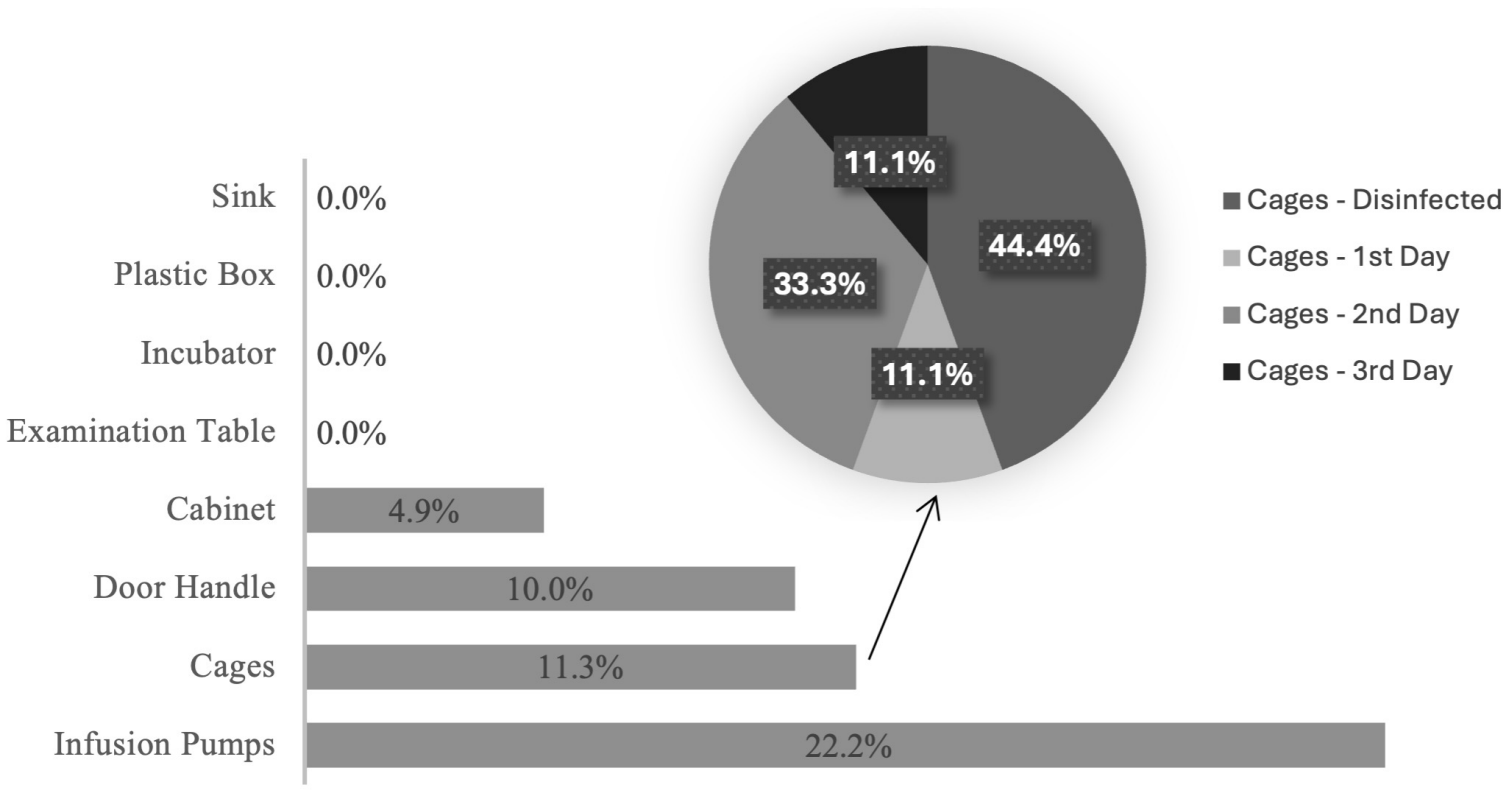
Proportion of positive samples obtained from each surface.

Considering all samples collected (*n* = 200), only 8% (*n* = 16/200) were positive for enterococcal presence. Infusion pumps were the surface that originated the highest relative number of positive samples (*n* = 4/18, 22.2%), followed by cages (*n* = 9/80, 11.3%), door handles (*n* = 1/10, 10.0%), and cabinets (*n* = 2/41, 4.9%); all other locations were negative for the presence of *Enterococcus* spp. (Figure 2). There were also more positive samples collected from dogs’ isolation rooms (*n* = 10/16, 62.5%) than cats’ isolation rooms (*n* = 6/16, 37.5%). No isolates were collected from cat cages during the disinfection process or from fully disinfected cages. However, it was possible to obtain 4 positive samples from fully disinfected dog cages, 1 from a cage on the first day of disinfection, 3 on the second day and 1 on the third day. Sampling procedure allowed to obtain a total of 50 presumptive enterococci.

### 3.2 *Enterococcus* identification and fingerprinting

**Figure 3:**
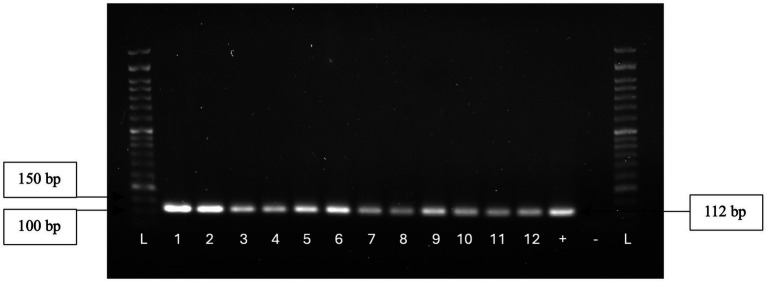
Genus identification of 12 isolates through the observation of a 112 bp amplicon.

A total of 73 isolates, 23 from 2019 and 50 from 2022, were submitted to PCR for isolates identification at genus and species levels. All isolates were confirmed as belonging to the *Enterococcus* genus (Figure 3), with 60 (82.2%) being identified as *E. faecium*, 9 (12.3%) as *E. hirae*, and 4 (5.5%) as *E. faecalis*.

**Figure 4:**
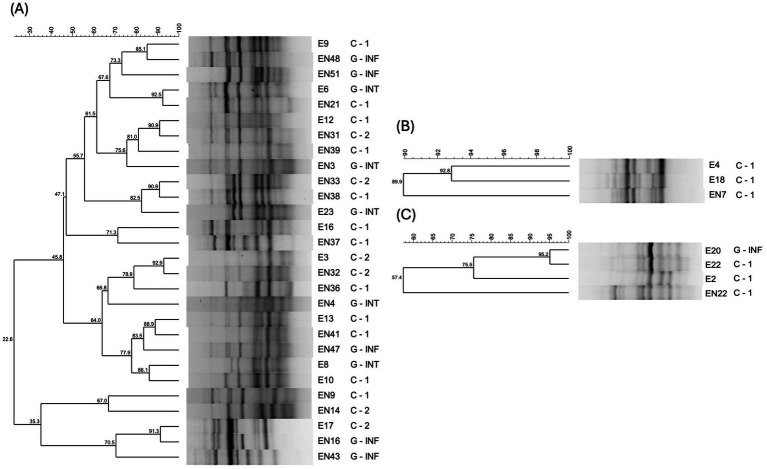
Dendrograms of representative *Enterococcus faecium* **(A)**, *Enterococcus faecalis* **(B)** and *Enterococcus hirae* **(C)** isolates, obtained using the (GTG), primer. Isolates from 2019 are represented by an E followed by a number and isolates from 2024 are represented by an EN followed by a number. The second row, following identification, represents place of collection for each isolate - C1: dogs’ hospitalization room 1; C2: dogs’ hospitalization room 2; G-INT: cats’ hospitalization room for intermediate patients; GINF: cats’ hospitalization room for infected patients.

Fingerprinting allowed for the selection of 34 representative isolates, 27 belonging to the species *E. faecium*, 4 to *E. hirae* and 3 to *E. faecalis*, which were further characterized. The gels and dendrogram used for selection can be found in Supplementary material 1. As seen in Figure 4, after this selection, it was possible to find isolates with a high similarity from samples collected in different years, more specifically E6 and EN21, E12 and EN31, E3 and EN32, and E17 and EN16.

### 3.3 Antimicrobial susceptibility testing

Antimicrobial susceptibility testing was accomplished through the disk diffusion method, allowing to classify isolates into three distinct phenotypes, according to the diameter of the inhibition halo produced: resistant, intermediate, and susceptible (Table 2).

**Table 2:** Results from the antimicrobial susceptibility tests of all isolates under study (*n* = 34), with corresponding susceptibility and resistance rates for each antibiotic tested.

	Isolates	AMP	AMC	CIP	LEV	TE	DO	ERY	VA	CN	STR
R (%)	*Enterococcus faecium* (*n* = 27)	24 (88.9)	24 (88.9)	19 (70.4)	16 (59.3)	18 (66.7)	3 (11.1)	26 (96.3)	0 (−)	4 (14.8)	5 (18.5)
	*Enterococcus hirae* (*n* = 4)	1 (25.0)	0 (−)	0 (−)	0 (−)	3 (75.0)	0 (−)	0 (−)	0 (−)	0 (−)	0 (−)
	*Enterococcus faecalis* (*n* = 3)	0 (−)	0 (−)	1 (33.3)	1 (33.3)	2 (66.7)	1 (33.3)	1 (33.3)	0 (−)	0 (−)	0 (−)
	**Total in 2019 (n = 14)**	10 (71.4)	9 (64.3)	4 (28.6)	3 (21.4)	12 (85.7)	4 (28.6)	8 (57.1)	0 (−)	4 (28.6)	2 (14.3)
	**Total in T 2022 (n = 20)**	15 (75.0)	15 (75.0)	16 (80.0)	14 (70.0)	11 (55.0)	0 (−)	19 (95.0)	0 (−)	0 (−)	3 (15.0)
	**Total (n = 34)**	25 (73.5)	24 (70.6)	20 (58.8)	17 (50.0)	23 (67.6)	4 (11.8)	27 (79.4)	0 (−)	4 (11.8)	5 (14.7)
I (%)	*Enterococcus faecium* (*n* = 27)	*	0 (−)	5 (18.5)	1 (3.7)	0 (−)	4 (14.8)	1 (3.7)	0 (−)	*	*
	*Enterococcus hirae* (*n* = 4)	*	0 (−)	3 (75.0)	0 (−)	0 (−)	0 (−)	0 (−)	0 (−)	*	*
	*Enterococcus faecalis* (*n* = 3)	*	0 (−)	2 (66.7)	0 (−)	1 (33.3)	1 (33.3)	2 (66.7)	2 (66.7)	*	*
	**Total in 2019 (n = 14)**	*	0 (−)	10 (71.4)	1 (7.1)	1 (7.1)	3 (21.4)	3 (21.4)	2 (14.3)	*	*
	**Total in 2022 (n = 20)**	*	0 (−)	0 (−)	0 (−)	0 (−)	2 (10.0)	0 (−)	0 (−)	*	*
	**Total (n = 34)**	*	0 (−)	10 (29.4)	1 (2.9)	1 (2.9)	5 (14.7)	3 (8.8)	2 (5.9)	*	*
**S (%)**	*Enterococcus faecium* (*n* = 27)	3 (11.1)	3 (11.1)	3 (11.1)	10 (37.0)	9 (33.3)	20 (74.1)	0 (−)	27 (100)	23 (85.2)	22 (81.5)
	*Enterococcus hirae* (*n* = 4)	3 (75.0)	4 (100)	1 (25)	4 (100)	1 (25.0)	4 (100)	4 (100)	4 (100)	4 (100)	4 (100)
	*Enterococcus faecalis* (*n* = 3)	3 (100)	3 (100)	0 (−)	2 (66.7)	0 (−)	1 (33.3)	0 (−)	1 (33.3)	3 (100)	3 (100)
	**Total in 2019 (n = 14)**	4 (28.6)	5 (35.7)	0 (−)	10 (71.4)	1 (7.1)	7 (50.0)	3 (21.4)	12 (85.7)	10 (71.4)	12 (85.7)
	**Total in 2022 (n = 20)**	5 (25.0)	5 (25.0)	4 (20.0)	6 (30.0)	9 (45.0)	18 (90.0)	1 (5.0)	20 (100)	20 (100)	17 (85.0)
	**Total (n = 34)**	9 (26.5)	10 (29.4)	4 (11.8)	16 (47.1)	10 (29.4)	25 (73.5)	4 (11.8)	32 (94.1)	30 (88.2)	29 (85.3)

AMP, ampicillin; AMC, amoxicillin-clavulanic acid; CIP, ciprofloxacin; LEV, levofloxacin; TE, tetracycline; DO, doxycycline; ERY, erythromycin; VA, vancomycin; CN, gentamicin; STR, streptomycin; R (%), number of resistant isolates and corresponding percentage; I (%), number of intermediate isolates and corresponding percentage; S (%), number of susceptible isolates and corresponding percentage. *No breakpoints available.

As observed, erythromycin was the antibiotic with a higher degree of resistance (*n* = 27/34, 79.4%), followed by ampicillin (*n* = 25/34, 73.5%), amoxicillin-clavulanic acid (*n* = 24/34, 70.6%), tetracycline (*n* = 23/34, 67.6%), ciprofloxacin (*n* = 20/34, 58.8%), levofloxacin (*n* = 17/34, 50.0%), streptomycin (*n* = 5/34, 14.7%), doxycycline (*n* = 4/34, 11.8%) and gentamicin (*n* = 4/34, 11.8%). All isolates were susceptible to chloramphenicol, linezolid and teicoplanin and no isolate was resistant to vancomycin.

Although resistance to high-level gentamicin and high-level streptomycin were similar, no isolates were resistant to both antibiotics simultaneously.

Moreover, 70.6% of the isolates were considered as MDR, i.e., resistant to one or more agents from three or more antimicrobial categories, excluding intrinsic resistances (31). Intermediate classifications were considered as susceptible for this characterization.

### 3.4 Virulence assays

As seen in Table 3, the majority of the enterococci isolated were capable of producing biofilm (*n* = 33/34, 97.1%) in Congo Red agar, and of producing hemolysin (*n* = 30/34, 88.2%). A total of 7 isolates (20.6%) were able to produce proteinase, and only 1 isolate (2.9%), identified as *E. faecalis*, was capable of producing gelatinase. No isolates produced either DNAse or lecithinase.

**Table 3:** Virulence profile of all isolates under study (*n* = 34).

		Hemolysin	Gelatinase	Biofilm (CR)	Proteinase
Positive (%)	*Enterococcus faecium* (*n* = 27)	27 (100)	0 (−)	26 (96.3)	4 (14.8)
	*Enterococcus hirae* (*n* = 4)	3 (75.0)	0 (−)	4 (100)	1 (25.0)
	*Enterococcus faecalis* (*n* = 3)	0 (−)	1 (33.3)	3 (100)	2 (66.7)
	**Total in 2019 (n = 14)**	12 (85.7)	1 (7.1)	13 (92.9)	6 (42.9)
	**Total in 2022 (n = 20)**	18 (90.0)	0 (−)	20 (100)	1 (5.0)
	**Total (n = 34)**	30 (88.2)	1 (2.9)	33 (97.1)	7 (20.6)
Negative (%)	*Enterococcus faecium* (*n* = 27)	0 (−)	27 (100)	1 (3.7)	23 (85.2)
	*Enterococcus hirae* (*n* = 4)	1 (25.0)	4 (100)	0 (−)	3 (75.0)
	*Enterococcus faecalis* (*n* = 3)	3 (100)	2 (66.7)	0 (−)	1 (33.3)
	**Total in 2019 (n = 14)**	2 (14.3)	13 (92.9)	1 (7.1)	8 (57.1)
	**Total in 2022 (n = 20)**	2 (10.0)	20 (100)	0 (−)	19 (95.0)
	**Total (n = 34)**	4 (11.8)	33 (97.1)	1 (2.9)	27 (79.4)

CR, Congo Red.

In terms of the optimization of glucose concentrations to be used in the biofilm quantification assay through crystal violet, the highest average OD values obtained for the 0.5% supplementation and the 1% supplementation were very similar for two isolates, with differences of 0.013 and 0.090. The third isolate, however, presented a higher difference (0.249), yielding higher OD values at a 0.5% supplementation. Additionally, for the first two isolates mentioned, the standard deviation was much lower in the assays using a 0.5% supplementation, associated with more consistent results among replicates. Therefore, the 0.5% supplementation was chosen for the remaining assays.

Regarding biofilm formation, evaluated through the crystal violet microtiter protocol, a total of 6 isolates (17.7%) were classified as non-producers, 22 (64.7%) as weak producers, 5 (14.7%) as moderate producers, and 1 (2.9%) as a strong producer.

Results regarding both the percentage of glucose supplementation optimization and crystal violet biofilm assays can be found in Supplementary material 2.

### 3.5 Pathogenicity potential—MAR and virulence indexes

The MAR and virulence indexes for the isolates evaluated in this study are presented in Table 4.

**Table 4:** MAR and virulence indexes and corresponding classification of each isolate.

ID	E2	E3	E4	E6	E8	E9	E12	E13	E16	E17	E18	E20
MAR	0.15	**0.46**	0.15	**0.46**	**0.46**	**0.46**	**0.38**	**0.46**	0.23	**0.54**	0.00	0.00
VIR	0.33	**0.50**	**0.50**	**0.50**	0.17	0.33	0.17	0.33	0.17	0.33	0.33	**0.50**
ID	E22	E23	EN3	EN4	EN7	EN9	EN14	EN16	EN21	EN22	EN31	EN32
MAR	0.08	**0.46**	**0.38**	**0.38**	**0.31**	**0.38**	**0.46**	**0.54**	**0.46**	0.08	**0.46**	**0.46**
VIR	0.33	0.33	0.33	0.33	0.17	0.33	0.33	0.33	**0.50**	0.17	0.33	0.33
ID	EN33	EN36	EN37	EN38	EN39	EN41	EN43	EN47	EN48	EN51		
MAR	**0.46**	0.08	0.08	**0.38**	**0.38**	**0.38**	0.08	**0.46**	**0.46**	**0.46**		
VIR	0.33	0.33	0.33	0.33	0.33	0.33	0.33	0.17	0.17	0.33		

E: 2019 Isolates; EN: 2022 Isolates. Bold values are the values above the cut – off.

The mean MAR index for all isolates was 0.34 (±0.17), while for isolates from 2019 was 0.31 (±0.20), and for isolates from 2022 was 0.36 (±0.15). The mean virulence index for all isolates was 0.32 (±0.10), while for isolates from 2019 was 0.35 (±0.12), and for isolates from 2022 was 0.31 (±0.08).

As described by Singh et al. (27), MAR and virulence indexes can be collectively used to classify the pathogenicity potential of the different isolates into four different threat categories: (i) high threat, if MAR index ≥0.30 and virulence index ≥0.50; (ii) moderate threat, if MAR index <0.30 and virulence index ≥0.50; (iii) low threat, if MAR index ≥0.30 and virulence index <0.50 and (iv) no threat, if MAR index <0.30 and virulence index <0.50. Based on this classification, from the totality of isolates presented in this study, 3 isolates (8.8%) were classified as high threats, 2 (5.9%) as moderate threats, 21 (61.8%) as low threats, and 8 (23.5%) as no threat.

### 3.6 Statistical analysis

The statistical analysis, detailed in Supplementary material 3, did not reveal any significant associations or differences between the variables under study. The majority of the Pearson’s chi-squared tests and Fisher’s exact tests originated high *p*-values (*p*-values >0.5), suggesting that the susceptibility and virulence patterns of the isolates remained consistent over time.

For individual antibiotics, no significant alterations were observed in resistance frequencies between isolates from Year 1 and Year 2. Similarly, the combined analysis of all antibiotics showed no overall change in the susceptibility patterns of the isolates. The analysis of virulence factors, both individually and combined, also indicated no significant differences between the 2 years. Additionally, the frequency of multidrug resistance (MDR) remained unchanged.

These results suggest a stable pattern in antibiotic susceptibility and virulence profiles of the isolates over the study period, with no strong relationship or dependency on the year of sampling. However, a larger sample size or more sensitive methods could potentially detect subtler changes or trends.

## 4 Discussion

Enterococci are regarded as bacteria of great importance in terms of antimicrobial resistance, with vancomycin-resistant *Enterococcus faecium* being considered by the World Health Organization (WHO) as a high-priority pathogen for the development of new antibiotics (32). These bacteria are also recognized for their high environmental permanence ability, presenting a noteworthy resistance to numerous adverse conditions, and a significant capacity for genome plasticity (3–6). All these characteristics make them important pathogens in terms of HAIs, so it is important to evaluate enterococcal presence and characterization in areas of high-risk transmission, such as hospitals (6).

The proportion of positive enterococcal samples obtained in this study in 2022 (8.0%) was similar to the one obtained in 2012 by Hamilton et al. (33) (9.5%) in the United States (US); however, it was much lower than the one obtained in 2023 by Singaravelu et al., (34) (27.4%) in Ireland. All three studies report samples obtained from veterinary hospitals; however, our samples were the only ones obtained strictly from an isolation unit. This lower proportion of positive samples was expected due to surface disinfection being a part of the strict protocols implemented for infection control in the BICU. However, when taking into account that a great proportion of animals, mainly dogs, admitted to this unit present some kind of gastrointestinal disease, as stated by Machado et al. (17), and that they are usually under antibiotic pressure, it is safe to admit that both these factors could lead to the selection of resistant bacteria (35–39), especially *Enterococcus* strains, since they present intrinsic resistance to many of the antibiotics regularly used in these treatments (4–6, 40).

Gastrointestinal diseases are usually associated with diarrhea, which consequently means that the cages that contain these patients are frequently contaminated with a higher degree of organic matter, rendering them harder to clean and disinfect. This hypothesis is supported by the fact that the second surface with a higher proportion of positive samples were dog cages. The higher proportion of positive samples in fully disinfected cages could be associated with the fact that enterococci are ubiquitous in the environment and could be present in materials used to clean these cages or could be vehiculated by the personnel responsible for cleaning or transporting them.

The higher proportion of positive samples collected from infusion pumps in this study could be due to the fact that, when samples were collected, these pumps were allocated to animals and sometimes, by lapse, may circumvent the disinfection process and serve as a source of cross-contamination.

In terms of ubiquitous bacteria such as *Enterococcus*, a simple routine evaluation of the proportion of positive/negative samples is not enough to determine the degree of concern that these surfaces represent in terms of infection control, and that further procedures, such as susceptibility tests, should be considered.

In regards to species prevalence, only Hamilton et al. (33) presented comparable results, with *E. faecium* also being the species with a higher percentage of representation in the samples analyzed by those authors. However, they also indicated that the second most prevalent species was *E. faecalis*, followed by *E. durans*, with no other species present. On the other hand, our study revealed a higher prevalence of *E. hirae*, followed by *E. faecalis*. In terms of isolates collected from cats and dogs, *E. faecium* and *E. faecalis* are usually the most prevalent species found, with some studies describing a higher percentage of *E. faecium* while others revealing a higher percentage of *E. faecalis* (41–47). Although some of these studies do not report the presence of *E. hirae* isolates in samples collected from animals, other studies, including the present one, describe a higher prevalence of this species in comparison to either *E. faecium* or *E. faecalis* (44, 47). Kataoka et al. (45) indicated a higher proportion of *E. hirae* in animals with no previous antibiotic exposure (i.e., less than 4 months old), whereas Ghosh et al. (48) and Jackson et al. (44) denoted that this could be the predominant species in cats. Contrarily to these, the majority of the isolates under study were collected from dog’s isolation rooms that frequently contain animals going through antibiotic treatment. However, if we consider that, in Kataoka et al.’s (45) study, the presence of *E. hirae* is associated with age rather than antibiotic exposure, the higher proportion of this species could be linked to the fact that the average age of dogs at the UICB is around 8 months (17).

The fingerprinting dendrogram obtained prior to the selection of the representative isolates, available in Supplementary material 1, shows that many isolates with high similarity were collected from the same room of the BICU. This is particularly evident for *E. hirae* and *E. faecalis* isolates, which all but one *E. hirae* and one *E. faecalis* isolate were originated from samples taken in the same room. However, exceptions exist, such as isolates E1 and E3, which, despite having a 91.4% similarity, were collected from different hospitalization rooms of the BICU. Similar cases include EN15 and EN23 (96.9% similarity), EN32 and EN40 (95.7%), and E6 and E15 (91.9%). Notably, E6 was collected from a hospitalization room for dogs, while E15 was originated from a hospitalization room for cats. This pattern could suggest potential cross-contamination between rooms. Given the high positivity rate of infusion pumps, which are one of the few items shared between all rooms, they could serve as a possible source of cross-contamination despite thorough disinfection.

Enterococci are known for their intrinsic and extrinsic resistance to a number of antibiotics (3–6). This is the reason why it is important to evaluate the antibiotic susceptibility of strains present in different critical environments such as isolation units, where they could possibly cause nosocomial infections in immune-suppressed animals (5).

The analyzed isolates presented a resistance rate of 73.5% to ampicillin, mainly represented by resistant *E. faecium* isolates (88.9%) (Table 2). This higher resistance ratio was also observed in a study performed in 2011 by Ghosh et al. (48) in the US, in which this species presented a 96.5% resistance to this antibiotic. These results do not come as a surprise, as it is well-known that *E. faecium* are usually more prone to *β*-lactam resistance (43, 47, 48), since they are known to frequently present the *pbp5* gene, which is the one most frequently associated with penicillin and cephalosporin resistance in *Enterococcus* (11, 49). On the other hand, when comparing the resistance rate observed in this study with others that evaluated isolates from veterinary clinics or hospitals’ surfaces or animals (cats and dogs), the value observed in this study is the second highest, surpassed only by Ghosh et al. (48). Other percentages found for total resistance are much lower (0.05–39.0%) (33, 41–43, 45, 47, 50, 51), with Iseppi et al. (46) being the only describing a resistance rate higher than 50% (56.5%). The resistance to amoxicillin-clavulanic acid (70.6%) found closely mirrored the one observed for ampicillin (73.5%), a finding consistent with the results reported by Awosile et al. (51). This similarity in resistance aligns with the understanding that *β*-lactamase production is rarely a mechanism associated with β-lactam resistance in enterococci (11). Both these resistance rates could be associated with the fact that amoxicillin-clavulanic acid is possibly the most frequent antibiotic used in the BICU.

Resistance to tetracycline (67.6%) was very similar to other studies performed in Portugal with isolates from animal origin (67.0–67.3%) (42, 50). Higher rates of tetracycline resistance in enterococci are not a novelty, as reported by various studies, which described values ranging between 43.0 and 97.4% (33, 34, 48, 50). Likewise, high rates of erythromycin resistance in enterococci have also been frequently reported elsewhere (41, 43, 46, 47).

Furthermore, resistance to both ciprofloxacin (58.8%) and levofloxacin (50.0%) were higher compared to findings in other studies, which reported resistance rates ranging from 7.8 to 57.7% for ciprofloxacin, and from 17.0 to 41.8% for levofloxacin (33, 43, 46, 47, 50), with the exception of those reported for ciprofloxacin by Rodrigues et al. (41) (73.1%) and Jackson et al. (44) (10–90%). Even though both compounds are not antibiotics used in the BICU, this higher resistance could be associated with the frequent use of other fluoroquinolones, such as enrofloxacin and marbofloxacin. Contrastingly, resistance to doxycycline, an antibiotic frequently used in veterinary medicine and in the BICU, and resistance to high-level gentamicin (11.8%) and high-level streptomycin (14.7%), were among the lowest reported (42, 44–48). Additionally, no isolate presented high-level resistance to both aminoglycosides.

No isolate presented vancomycin, linezolid or teicoplanin resistance, which has already been described in similar studies on veterinary settings (33, 41–43, 45, 46, 48). On the other hand, the fact that no isolates were resistant to chloramphenicol was not expected, as the majority of studies reported isolates resistant to this antibiotic (42, 44–46, 50–52); however, this lack of resistance could be explained by the fact that chloramphenicol is not routinely used in this isolation unit. The absence of isolates resistant to these compounds can be considered as positive, since vancomycin-resistant enterococci have been a rising threat in today’s medicine (32), and chloramphenicol has been effectively used in human medicine, in association with other antibiotics, against infections promoted by these bacteria (53, 54).

Virulence factors represent an advantageous feature of bacteria in terms of environmental survival. For example, they can be associated with adherence to a variety of surfaces and biofilm formation (11), which could impair the disinfection process (55, 56). This becomes especially problematic considering that the vast majority of the isolates from this study were found to be positive for biofilm production, according to both the Congo Red agar (97.1%) and Crystal Violet (82.3%) methodologies. The occurrence of this virulence factor in high rates in *Enterococcus* isolates has been reported in other studies (29, 30, 57, 58), and may explain the detection of isolates with similar fingerprinting profiles in samples collected 3 years apart (Figure 4). Notably, seven of those eight isolates (excluding E12) demonstrated biofilm-forming ability in crystal violet assays. Furthermore, it is concerning that this group of similar isolates from different years also includes all three isolates characterized as high threats (E3, E6 and EN21), as they could potentially serve as sources for the dissemination of resistance and virulence determinants.

Similarly, hemolysin production ability was also frequently detected in the isolates under study (88.2%). Contrarily to biofilm formation, the prevalence of this virulence factor reported in different studies is more variable. Analogously to our study, Iseppi et al. (46) reported hemolysin production rates of 63.5% while Poeta et al. (59) reported approximately 22% of positivity for hemolysin production. Cunha et al. (58) reported between 56 and 75%, however in this study *α*-haemolysis was considered as a negative result, contrarily to the present report and the other mentioned studies (46, 59). This variation could be associated with isolate’s origin, since this study was the only one that analyzed isolates from samples majorly collected from surfaces associated with non-healthy animals, notwithstanding possible human-origin contaminations.

On the other hand, the presence of gelatinase-positive isolates was lower in the present study (2.9%) in comparison to others (8.7–46.9%) (46, 58–60). The only gelatinase-positive isolate found was identified as *E. faecalis*, which seems to be the most frequent species associated with this virulence factor (46, 59, 60). Taking this into account, the lower percentage of gelatinase-positive isolates observed could be associated with the low proportion of representativity of this species in the totality of the isolates’ collection under study.

Considering the work developed by Verdial et al. (19), a new bacteriological control plan with optimized disinfection protocols was implemented between both sampling periods mentioned in this study. As such, this study also aimed to evaluate the statistical significance between the variations of the resistance profiles of the isolates collected in 2019 and 2022. This analysis aimed to determine any possible correlations between variation in disinfection protocols and antibiotic resistance, however, it was not possible to detect any significant associations or differences between the variables, indicating that there may not be a strong relationship or dependency between isolates’ resistance profiles and the year of the sampling, and, therefore, with the disinfection protocol under practice. However, it is important to note that further research with larger sample sizes or alternative analytical approaches could be valuable to confirm these findings.

In the 2022 samples, the surface associated with isolates with a higher MAR index (Table 4) were cages sampled on the third day of the disinfection procedure (0.46), followed by cabinets (0.43), door handles (0.38), infusion pumps (0.37), disinfected cages (0.35), cages sampled on the first day of the disinfection procedure (0.27), and finally cages sampled on the second day of the disinfection (0.21). VIR indexes were similar for isolates from every surface (approximately 0.33), except for disinfected cages and infusion pumps, which were slightly lower (0.25). The only isolate presenting a high pathogenetic potential was obtained from a cage sampled on the first day of disinfection. Higher index rates for cabinets and door handles are of concern, since these could be perceived as higher risk for cross contamination, and consequently of higher risk for resistant determinants dissemination. The higher index for isolates from disinfected cages compared to those obtained from cages sampled on the first and second days of disinfection is also worrisome, since these cages are considered ready for receiving new patients.

One of the limitations of this article is associated with the high variability inherent in the fingerprinting technique. While this method can be used to effectively reduce the number of isolates in analysis, it can only be indicative of other information such as potential cross-contaminations between rooms, which remain speculative without further validation. Since confirming the occurrence of cross-contaminations was not the primary focus of this study, no additional verification methods were employed. Moreover, the virulence profile of each isolate was determined using phenotypic methods, which could be further validated and enhanced through molecular techniques. However, since virulence in enterococci is predominantly linked to *E. faecium* (11), the least represented species in this study with only three isolates, and given the constraints associated with the development of molecular techniques, no additional testing was conducted.

## 5 Conclusion

To the best of our knowledge, this is the first study that describes antibiotic resistance and virulence profiles of *Enterococcus* collected from a veterinary BICU. The classification of the majority of isolates as MDR, the high percentage of biofilm and hemolysin producers, and the isolation of three high-threat pathogens from this isolation unit, could be associated with the high number of animals with gastrointestinal disease and under antibiotic pressure, leading to a shift in the intestinal bacterial environment and selection of resistant microbial strains. This means that a regular monitorization of this kind of microorganisms, including not only their isolation but also their characterization in terms of resistance and virulence profiles, in these units and in veterinary hospitals and clinics, seems to be imperative in order to prevent the spread of these pathogens and the possible development of hospital associated infections.

## Data Availability

The original contributions presented in the study are included in the article/Supplementary material, further inquiries can be directed to the corresponding author/s.

## Supplementary material

SUPPLEMENTARY MATERIAL 1 Fingerprinting gels and corresponding dendrogram.

SUPPLEMENTARY MATERIAL 2 Glucose optimization protocol results and crystal violet assay results.

SUPPLEMENTARY MATERIAL 3 Statistical analysis report.
