# Supplementary material for: Pathogenicity potential of enterococci isolated from a Veterinary Biological Isolation and Containment Unit
Source: Front Vet Sci. 2024 Oct 21;11:1458069. doi: 10.3389/fvets.2024.1458069 (PMC11532069; doi:10.3389/fvets.2024.1458069)

Supplementary Materials 1 – Fingerprinting Gels

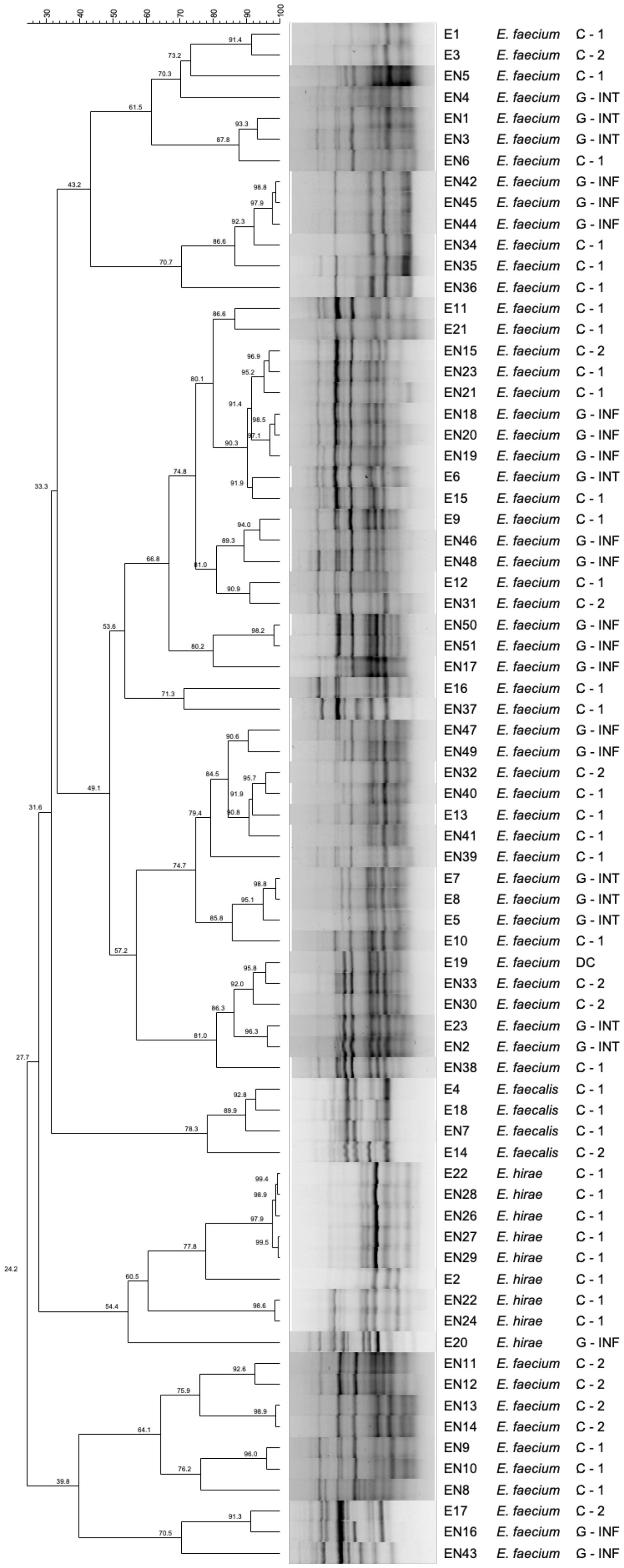

C1 and C2: Dogs' hospitalization rooms;

G – INT: Cats' hospitalization room for intermediate patients;

G – INF: Cats' hospitalization room for infected patients.

Supplementary Materials 1 – Fingerprinting Gels

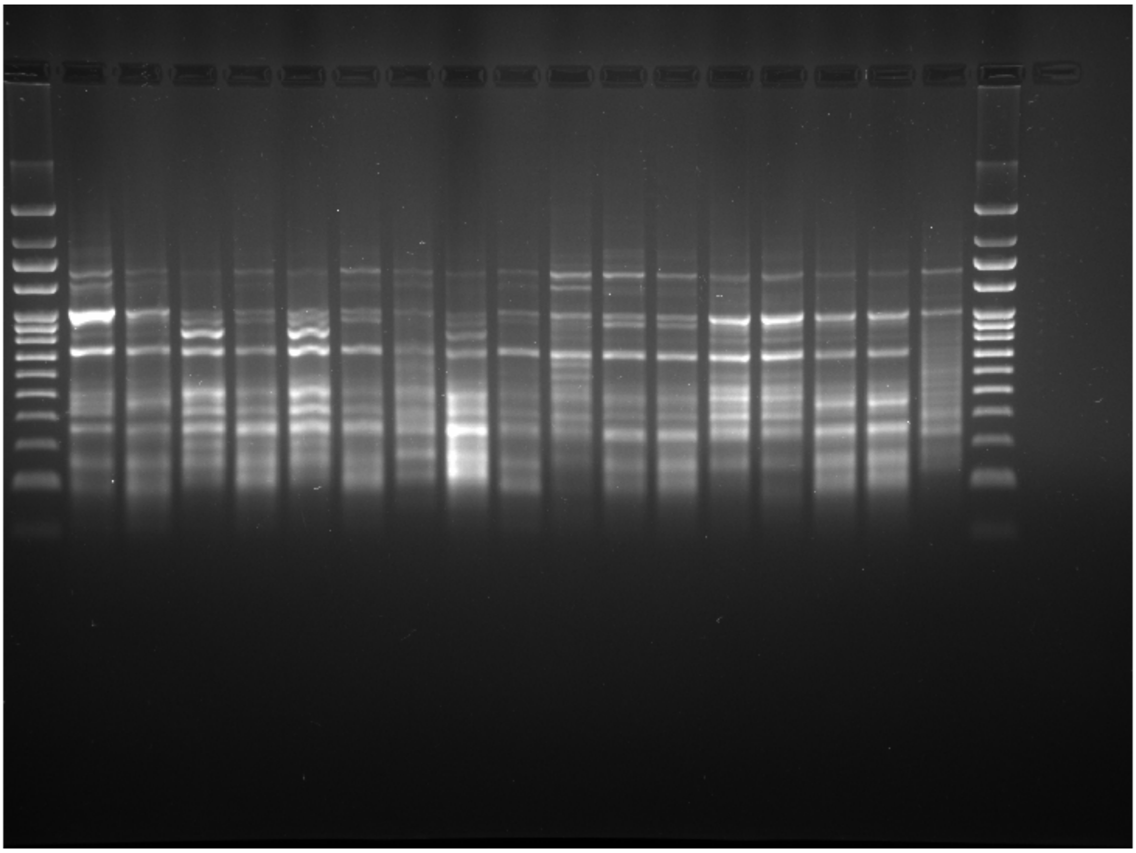

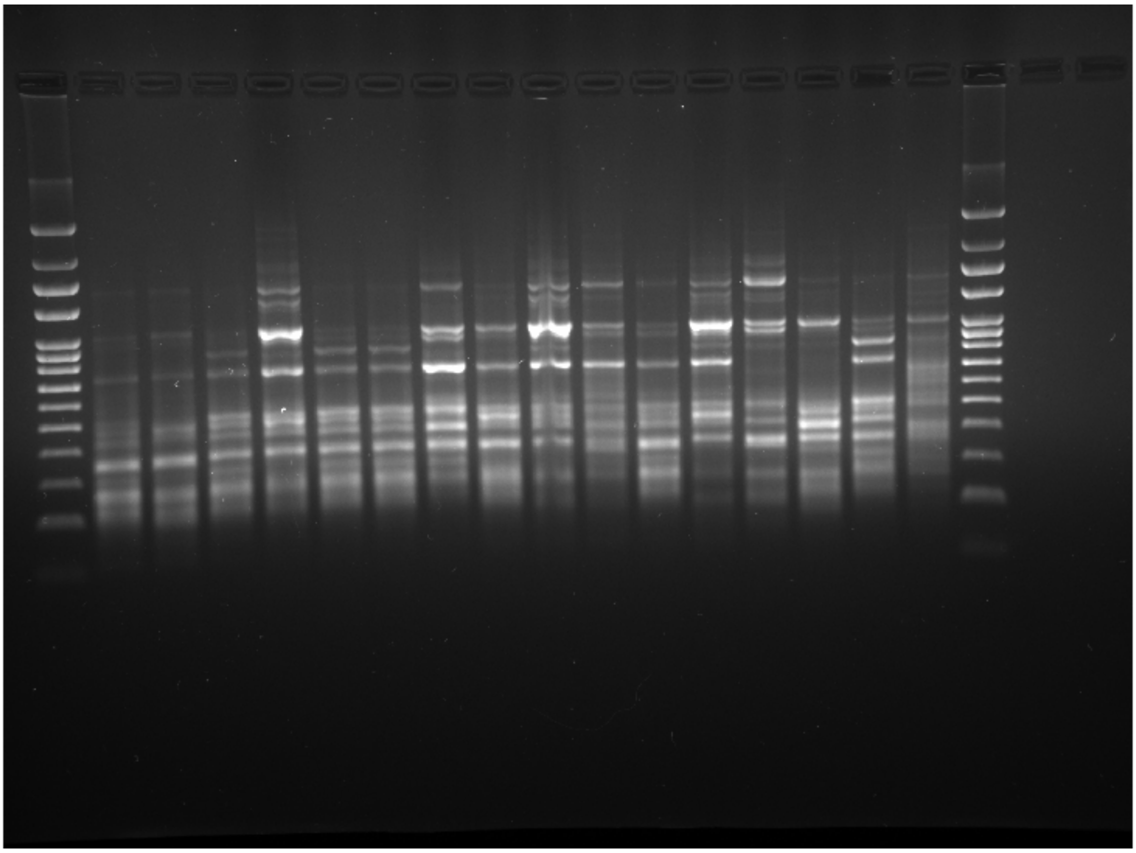

Supplementary Materials 1 – Fingerprinting Gels

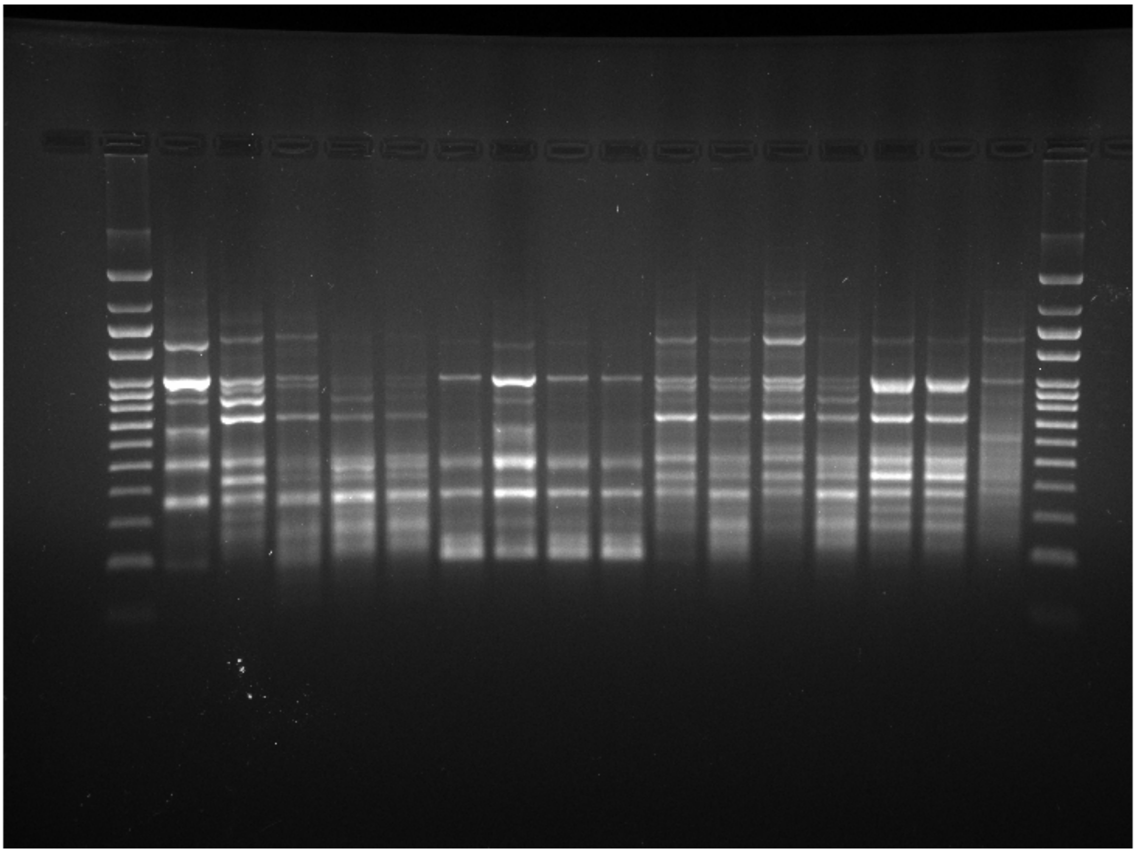

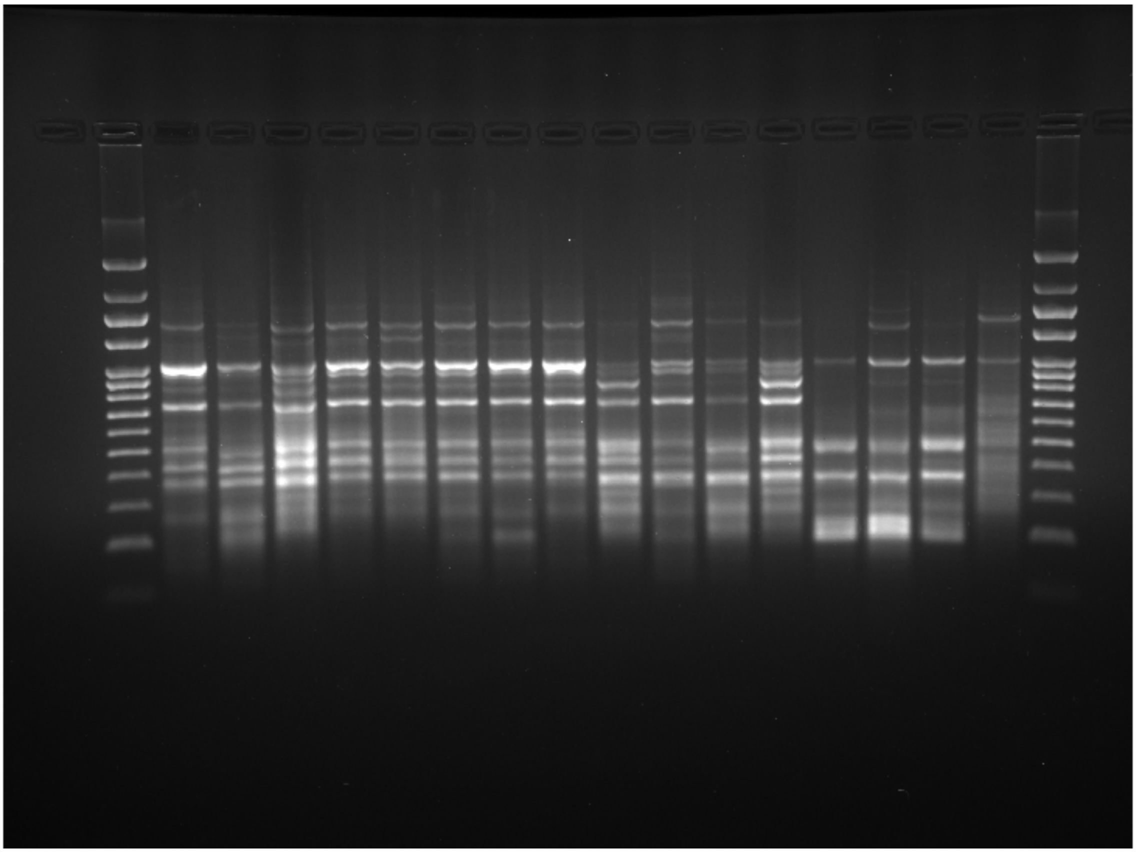

Supplementary Materials 1 – Fingerprinting Gels

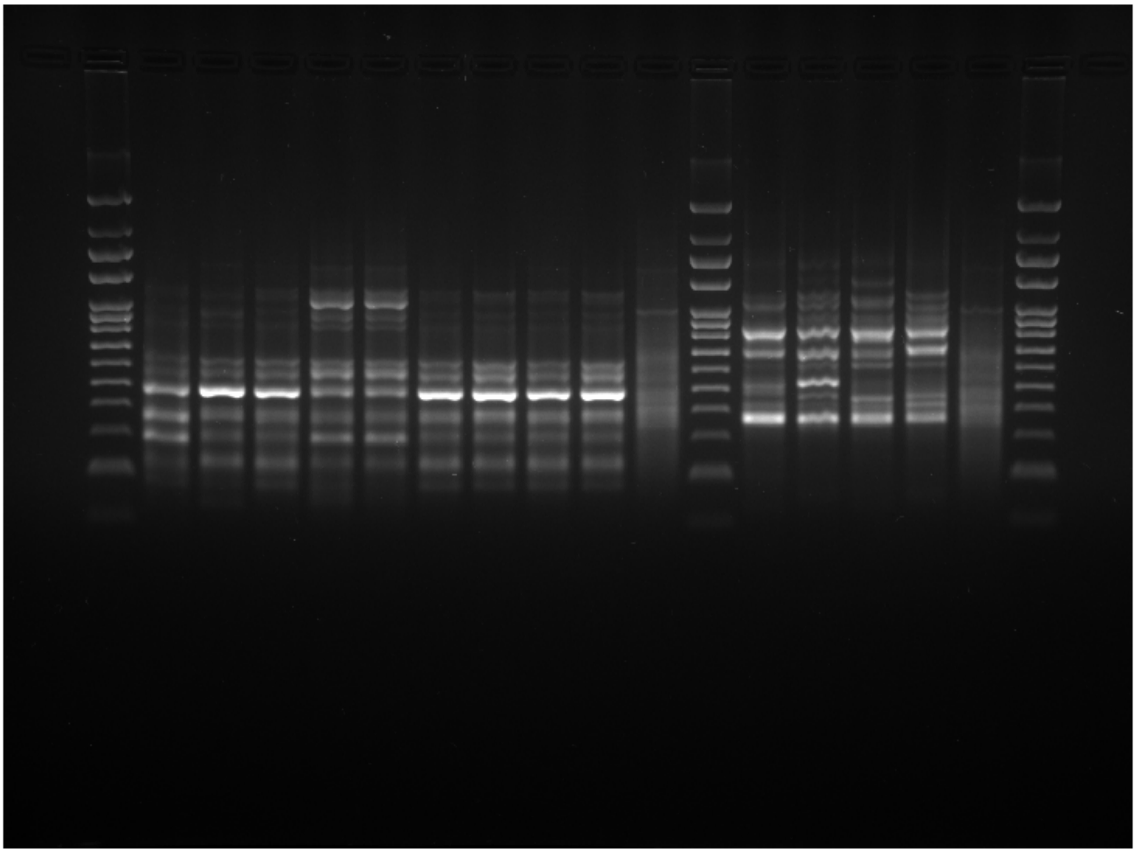

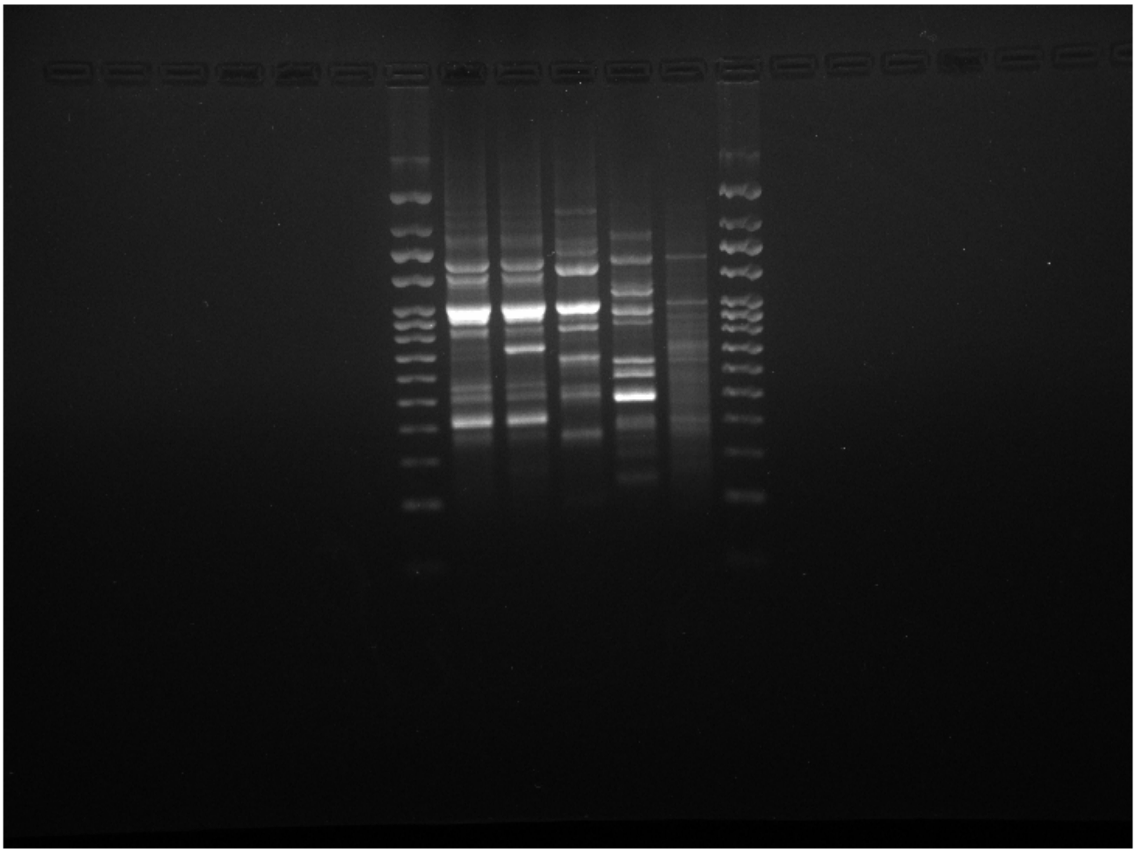

Supplement: SUPPLEMENTARY MATERIAL 1 — Fingerprinting gels and corresponding dendrogram. [file Data_Sheet_1.PDF]
