# Supplementary material for: Pathogenicity potential of enterococci isolated from a Veterinary Biological Isolation and Containment Unit
Source: Front Vet Sci. 2024 Oct 21;11:1458069. doi: 10.3389/fvets.2024.1458069 (PMC11532069; doi:10.3389/fvets.2024.1458069)
Supplement: SUPPLEMENTARY MATERIAL 2 — Glucose optimization protocol results and crystal violet assay results. [file Data_Sheet_2.PDF]

Supplementary Materials 2 - Crystal Violet Optical Density Readings and Averages

	1	2	3	4	5	6	7	8	9	10	11	12
A	0.28	0.628		0.365	0.556		0.37	0.742			0.221	0.218
B	0.199	0.674		0.299	1.618		0.274	0.710			0.190	0.433
C	0.182	0.734		0.342	1.169		0.282	0.694			0.194	0.205
D												
E												
F	0.32	0.207		1.159	0.249		0.385	0.344			0.185	0.172
G	0.525	0.176		1.189	0.178		0.262	0.480			0.221	0.196
H	0.344	0.181		0.870	0.214		0.399	0.492			0.189	0.169

	1	2	3	4	5	6	7	8	9	10	11	12
A	0.402	0.172		0.838	1.555		0.201	0.259			0.164	0.208
B	0.422	0.193		0.559	0.329		0.162	0.213			0.164	0.179
C	0.418	0.177		1.177	0.414		0.182	0.269			0.179	0.204
D												
E												
F	0.432	0.319		1.861	1.465		0.247	0.216			0.167	0.186
G	0.488	0.432		1.644	0.699		0.187	0.210			0.170	0.179
H	0.85	0.331		1.173	1.240		0.44	0.221			0.155	0.181

	1	2	3	4	5	6	7	8	9	10	11	12
A	0.289	0.556		1.108	0.193		0.384	0.188			0.214	0.204
B	0.24	0.466		1.587	0.228		0.496	0.197			0.184	0.186
C	0.29	0.821		1.849	0.262		0.438	0.197			0.336	0.178
D												
E												
F	0.341	0.190		0.217	0.224		0.207	0.216			0.238	0.200
G	0.42	0.194		0.226	0.231		0.221	0.203			0.288	0.325
H	0.586	0.185		0.231	0.213		0.303	0.219			0.239	0.169

Glucose Optimization - Optical Density Readings and Averages

Supplementary Materials 2 - Crystal Violet Optical Density Readings and Averages

0.25%

		1st Assay		2nd Assay		3rd Assay		FINAL	
ID		Average	SD	Average	SD	Average	SD	Average	SD
E. faecium	E12	0.220	0.052	0.414	0.011	0.273	0.029	0.302	0.092
E. hirae	E20	0.335	0.034	0.858	0.309	1.515	0.376	0.903	0.567
E. faecium	EN47	0.309	0.053	0.182	0.020	0.439	0.056	0.310	0.118

0.5%

		1st Assay		2nd Assay		3rd Assay		FINAL	
ID		Average	SD	Average	SD	Average	SD	Average	SD
E. faecium	E12	0.396	0.112	0.590	0.227	0.449	0.125	0.547	0.315
E. hirae	E20	1.073	0.176	1.559	0.352	0.225	0.007	0.952	0.617
E. faecium	EN47	0.349	0.075	0.291	0.132	0.244	0.052	0.295	0.092

1.00%

		1st Assay		2nd Assay		3rd Assay		FINAL	
ID		Average	SD	Average	SD	Average	SD	Average	SD
E. faecium	E12	0.679	0.053	0.181	0.011	0.614	0.614	0.491	0.254
E. hirae	E20	1.114	0.533	0.766	0.685	0.228	0.035	0.703	0.582
E. faecium	EN47	0.715	0.024	0.247	0.030	0.194	0.005	0.385	0.249

1.5%

		1st Assay		2nd Assay		3rd Assay		FINAL	
ID		Average	SD	Average	SD	Average	SD	Average	SD
E. faecium	E12	0.188	0.017	0.361	0.062	0.190	0.005	0.246	0.092
E. hirae	E20	0.214	0.036	1.135	0.394	0.223	0.009	0.524	0.499
E. faecium	EN47	0.439	0.082	0.216	0.006	0.213	0.009	0.289	0.120

SD = Standard Deviation

Glucose Optimization - Optical Density Readings and Averages

Supplementary Materials 2 - Crystal Violet Optical Density Readings and Averages

	1	2	3	4	5	6	7	8	9	10	11	12
**A**	0.269		0.292		0.203		0.191		0.199			
**B**	0.285		0.277		0.186		0.181		0.199			
**C**	0.364		0.331		0.214		0.245		0.201			
**D**												
**E**												
**F**	0.248		0.232		0.190		0.190		0.241			0.235
**G**	0.222		0.313		0.204		0.183		0.310			0.245
**H**	0.328		0.393		0.290		0.369		0.487			0.194

	1	2	3	4	5	6	7	8	9	10	11	12
**A**	0.287		0.615		0.365		0.213		0.286			
**B**	0.443		1.016		0.210		0.248		0.214			
**C**	0.401		0.850		0.223		0.221		0.225			
**D**												
**E**												
**F**	0.255		0.333		0.327		0.216		0.295			0.192
**G**	0.296		0.307		0.227		0.245		0.280			0.211
**H**	0.384		0.457		0.252		0.364		0.337			0.224

	1	2	3	4	5	6	7	8	9	10	11	12
**A**	0.303		0.458		0.269		0.196		0.462			
**B**	0.250		0.597		0.212		0.182		0.205			
**C**	0.300		0.584		0.311		0.216		0.210			
**D**												
**E**												
**F**	0.330		0.304		0.243		0.208		0.286			0.334
**G**	0.292		0.396		0.217		0.248		0.293			0.275
**H**	0.330		0.877		0.444		0.310		0.911			0.333

Biofilm Quantification Through Crystal Violet - Optical Density Readings and Averages

Supplementary Materials 2 - Crystal Violet Optical Density Readings and Averages

		1st Assay	2nd Assay	3rd Assay	Average	SD
E. hirae	E2	0.306	0.377	0.284	0.322	0.065
E. faecium	E3	0.266	0.312	0.317	0.298	0.051
E. faecalis	E4	0.300	0.827	0.546	0.558	0.253
E. faecium	E6	0.313	0.366	0.526	0.401	0.190
E. faecium	E8	0.201	0.266	0.264	0.244	0.059
E. faecium	E9	0.228	0.269	0.301	0.266	0.079
E. faecium	E12	0.206	0.227	0.198	0.210	0.025
E. faecium	E13	0.247	0.275	0.255	0.259	0.072
E. faecium	E16	0.200	0.242	0.292	0.245	0.086
E. faecium	E17	0.346	0.304	0.497	0.382	0.210
	C-	0.225	0.209	0.314	0.249	0.054

OD < ODc denoted as non-biofilm producers,

ODc < OD < 2ODc denoted as weak biofilm producers

2ODc < OD < 4ODc denoted as moderate biofilm producers

OD > 4ODc denoted as strong biofilm producers

Odc	0.249
2ODc	0.498
4ODc	0.997

Supplementary Materials 2 - Crystal Violet Optical Density Readings and Averages

	1	2	3	4	5	6	7	8	9	10	11	12
A	0.828	0.702		0.556		3.173		0.324	0.46			
B	1.678	0.944		0.348		3.356		0.28	0.302			
C	2.054	1.577		0.511		2.443		0.292	0.276			
D												
E												
F	0.523	0.25		0.368		0.401		0.585	1.785			0.325
G	0.541	0.271		0.591		0.45		0.764	1.996			0.31
H	0.564	0.254		0.612		0.757		1.513	1.972			0.296

	1	2	3	4	5	6	7	8	9	10	11	12
A	0.248	0.686		0.19		2.868		0.322	0.207			
B	0.364	0.615		0.212		1.658		0.287	0.186			
C	0.471	0.563		0.231		2.881		0.374	0.239			
D												
E												
F	0.284	0.242		0.255		0.225		0.207	0.428			0.202
G	0.342	0.215		0.264		0.238		0.266	0.444			0.219
H	0.32	0.301		0.266		0.263		0.334	0.323			0.209

	1	2	3	4	5	6	7	8	9	10	11	12
A	0.411	0.776		0.795		2.89		0.738	0.26			
B	0.659	0.647		0.272		2.258		0.243	0.22			
C	0.56	0.676		0.278		3.762		0.215	0.238			
D												
E												
F	0.403	0.212		0.255		0.281		0.564	0.526			0.254
G	0.334	0.25		0.362		0.315		0.321	0.575			0.305
H	0.344	0.296		0.588		0.306		0.474	0.685			0.228

Biofilm Quantification Through Crystal Violet - Optical Density Readings and Averages

Supplementary Materials 2 - Crystal Violet Optical Density Readings and Averages

		1st Assay	2nd Assay	3rd Assay	Average	SD
E. faecalis	E18	1.520	0.361	0.543	0.808	0.630
E. hirae	E20	0.543	0.315	0.360	0.406	0.107
E. hirae	E22	1.074	0.621	0.700	0.798	0.312
E. faecium	E23	0.258	0.253	0.253	0.255	0.031
E. faecium	EN3	0.472	0.211	0.448	0.377	0.203
E. faecium	EN4	0.524	0.262	0.402	0.396	0.157
E. faecalis	EN7	2.991	2.469	2.970	2.810	0.624
E. faecium	EN9	0.536	0.242	0.301	0.360	0.166
E. faecium	EN14	0.299	0.328	0.399	0.342	0.156
E. faecium	EN16	0.954	0.269	0.453	0.559	0.400
E. faecium	EN21	0.346	0.211	0.239	0.265	0.081
E. hirae	EN22	1.918	0.398	0.595	0.970	0.720
	C-	0.310	0.210	0.262	0.261	0.048

OD < ODc denoted as non-biofilm producers,

ODc < OD < 2ODc denoted as weak biofilm producers

2ODc < OD < 4ODc denoted as moderate biofilm producers

OD > 4ODc denoted as strong biofilm producers

ODc	0.261
2ODc	0.522
4ODc	1.044

Supplementary Materials 2 - Crystal Violet Optical Density Readings and Averages

	1	2	3	4	5	6	7	8	9	10	11	12
A	0.335	0.31		0.245		0.313		0.571	0.23			
B	0.263	0.245		0.481		0.308		0.222	0.21			
C	0.264	0.279		0.259		0.285		0.266	0.265			
D												
E												
F	0.258	0.291		0.264		0.298		0.273	0.418			0.338
G	0.252	0.27		0.251		0.314		0.256	0.315			0.344
H	0.3	0.317		0.373		0.444		0.304	0.338			0.316

	1	2	3	4	5	6	7	8	9	10	11	12
A	0.364	0.316		0.322		0.385		0.351	0.349			
B	0.28	0.24		0.22		0.279		0.208	0.368			
C	0.401	0.294		0.268		0.279		0.314	0.252			
D												
E												
F	0.314	0.253		0.281		0.274		0.252	0.343			0.23
G	0.349	0.228		0.354		0.321		0.242	0.372			0.219
H	0.44	0.209		0.315		0.503		0.279	0.399			0.238

	1	2	3	4	5	6	7	8	9	10	11	12
A	0.35	0.386		0.38		0.4		0.207	0.224			
B	0.341	0.342		0.254		0.277		0.187	0.21			
C	0.355	0.366		0.33		0.271		0.252	0.255			
D												
E												
F	0.381	0.294		0.314		0.349		0.251	0.295			0.217
G	0.367	0.281		0.276		0.302		0.243	0.299			0.277
H	0.514	0.533		0.433		0.438		0.359	0.376			0.28

Biofilm Quantification Through Crystal Violet - Optical Density Readings and Averages

Supplementary Materials 2 - Crystal Violet Optical Density Readings and Averages

		1st Assay	2nd Assay	3rd Assay	Average	SD
E. faecium	EN31	0.287	0.348	0.349	0.328	0.048
E. faecium	EN32	0.270	0.368	0.421	0.353	0.085
E. faecium	EN33	0.278	0.283	0.365	0.309	0.050
E. faecium	EN36	0.293	0.230	0.369	0.297	0.095
E. faecium	EN37	0.328	0.270	0.321	0.307	0.082
E. faecium	EN38	0.296	0.317	0.341	0.318	0.059
E. faecium	EN39	0.302	0.314	0.316	0.311	0.049
E. faecium	EN41	0.352	0.366	0.363	0.360	0.081
E. faecium	EN43	0.353	0.291	0.215	0.286	0.119
E. faecium	EN47	0.278	0.258	0.284	0.273	0.038
E. faecium	EN48	0.235	0.323	0.230	0.263	0.058
E. faecium	EN51	0.357	0.371	0.323	0.351	0.044
	C-	0.333	0.229	0.258	0.273	0.050

OD < ODc denoted as non-biofilm producers,

ODc < OD < 2ODc denoted as weak biofilm producers

2ODc < OD < 4ODc denoted as moderate biofilm producers

OD > 4ODc denoted as strong biofilm producers

ODc	0.273
2ODc	0.546
4ODc	1.093

Biofilm Quantification Through Crystal Violet - Optical Density Readings and Averages
