# Supplementary material for: Pathogenicity potential of enterococci isolated from a Veterinary Biological Isolation and Containment Unit
Source: Front Vet Sci. 2024 Oct 21;11:1458069. doi: 10.3389/fvets.2024.1458069 (PMC11532069; doi:10.3389/fvets.2024.1458069)
Supplement: SUPPLEMENTARY MATERIAL 3 — Statistical analysis report. [file Data_Sheet_3.PDF]

Supplementary Materials 3 - Statistical Analysis

1. TSA comparation Qualitative (R = Resistant, S = Susceptible, I = Intermediate)

1.1. Antibiotic individually comparing first year to second year

- The analysis involved calculating relative frequencies, creating a data frame to store the results, and performing statistical tests for each antibiotic. For each antibiotic, the frequencies of "R," "S," and "I" were calculated for both Year1 and Year2. Subsequently, Pearson's chi-squared tests and Fisher's exact tests were performed to assess the independence of susceptibility patterns between the two years.
- The results, presented below, include p-values from both chi-squared and Fisher's exact tests (The chi-squared test applies an approximation assuming the sample is large, while the Fisher's exact test runs an exact procedure especially for small-sized samples):
  - Ampicillin: p-value = 0.9545 (Chi-squared), 1 (Fisher's exact)
  - Amoxicillin-Clavulanic Acid: p-value = 0.8691 (Chi-squared), 1 (Fisher's exact)
  - Ciprofloxacin: p-value = 0.5605 (Chi-squared), 1 (Fisher's exact)
  - Levofloxacin: p-value = 0.7793 (Chi-squared), 1 (Fisher's exact)
  - Tetracycline: p-value = 0.8133 (Chi-squared), 1 (Fisher's exact)
  - Doxycycline: p-value = 0.8019 (Chi-squared), 1 (Fisher's exact)
  - Erythromycin: p-value = 0.8144 (Chi-squared), 1 (Fisher's exact)
  - Chloramphenicol: Chi-squared test inconclusive
  - Linezolid: Chi-squared test inconclusive
  - Vancomycin: p-value = 0.6949 (Chi-squared), 1 (Fisher's exact)
  - Teicoplanin: Chi-squared test inconclusive
  - Gentamicin: p-value = 0.5637 (Chi-squared), 1 (Fisher's exact)
  - Streptomycin: p-value = 0.9886 (Chi-squared), 1 (Fisher's exact)
- The results indicate that there were no significant changes between Year1 and Year2. However, it is essential to note inconclusive results for some antibiotics, where the chi-squared test did not provide a definitive assessment.

1.2. All Antibiotics comparing first and second year

- The analysis involved calculating relative frequencies, creating a data frame to store the results, and performing statistical tests for all the antibiotics of each year. For each year, the frequencies of "R," "S," and "I" were calculated. Subsequently, Pearson's chi-squared tests and Fisher's exact tests were performed to assess the independence of susceptibility patterns between the two years.
- The objective of the analysis was to scrutinize if there was a significant alteration in susceptibility frequency across the two years, without including the factor Antibiotic, i.e. different antibiotics.
- P-value = 0.9537 (Chi-squared), 1 (Fisher's exact) – No statistical difference was observed.

2. Virulence Factors comparation Qualitative ((+) and (-))

2.1. Virulence Factors Individually comparing first year to second year

- The analysis involved calculating relative frequencies, creating a data frame to store the results, and performing statistical tests for each virulence factor. Relative frequencies of each factor were calculated for both years, and statistical tests, including Pearson's Chi-squared test and Fisher's exact test, were conducted to assess the significance of observed changes.
- The results, presented below, include p-values from both chi-squared and Fisher's exact tests:

- Hemolysin: p-value = 0.9261(Chi-squared); 1 (Fisher's exact)
- Gelatinase: p-value = 0.7855 (Chi-squared); 1 (Fisher's exact)
- Biofilm (Congo Red): p-value = 0.7855 (Chi-squared); 1 (Fisher's exact)
- Biofilm (Cristal Violet): p-value = 0.7393 (Chi-squared); 1 (Fisher's exact)
- DNAse: Chi-squared test inconclusive
- Proteinase: p-value = 0.5304 (Chi-squared); 1 (Fisher's exact)
- Lecitinase: Chi-squared test inconclusive

  The results indicate that there were no significant changes between Year1 and Year2.

2.2. <u>All Virulence Factors comparing first and second year</u>

- The analysis involved calculating relative frequencies, creating a data frame to store the results, and performing statistical tests for all the virulence factors of each year. For each year, the frequencies of (+) and (-) were calculated. Subsequently, Pearson's chi-squared test and Fisher's exact test were used to evaluate any significant changes between the two years. The p-values obtained from these tests helped evaluate the significance of any observed changes.
- P-value (with Congo Red results) = 0.934 (Chi-squared), 1 (Fisher's exact) – No statistical difference was observed.
- P-value (with Cristal Violet results) = 0.9556 (Chi-squared), 1 (Fisher's exact) – No statistical difference was observed.

- **MDR**
- The analysis involved calculating relative frequencies, creating a data frame to store the results, and performing statistical tests for all the MDR isolates of each year. For each year, the frequencies of presence of MDR or not were calculated. Subsequently, Pearson's chi-squared test and Fisher's exact test were used to evaluate any significant changes between the two years. The p-values obtained from these tests helped evaluate the significance of any observed changes.
- P-value = 1 (Chi-squared), 1 (Fisher’s exact). No statistical difference was observed.
